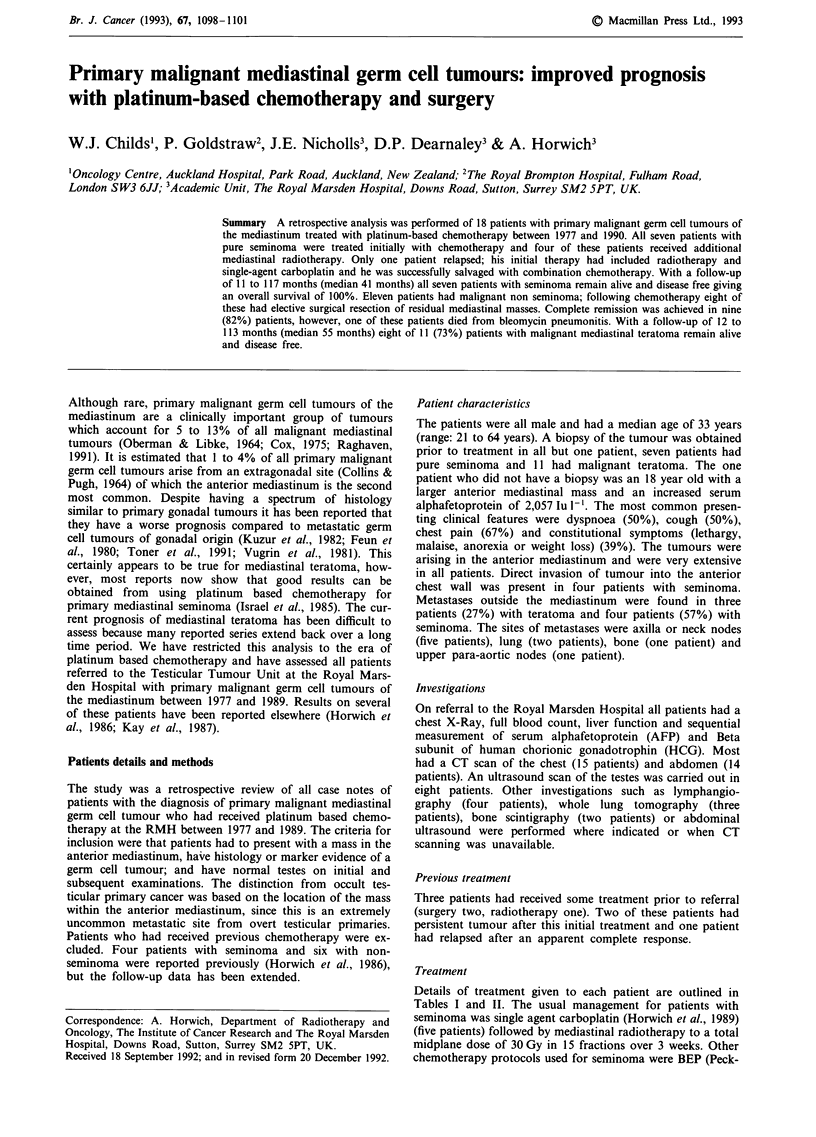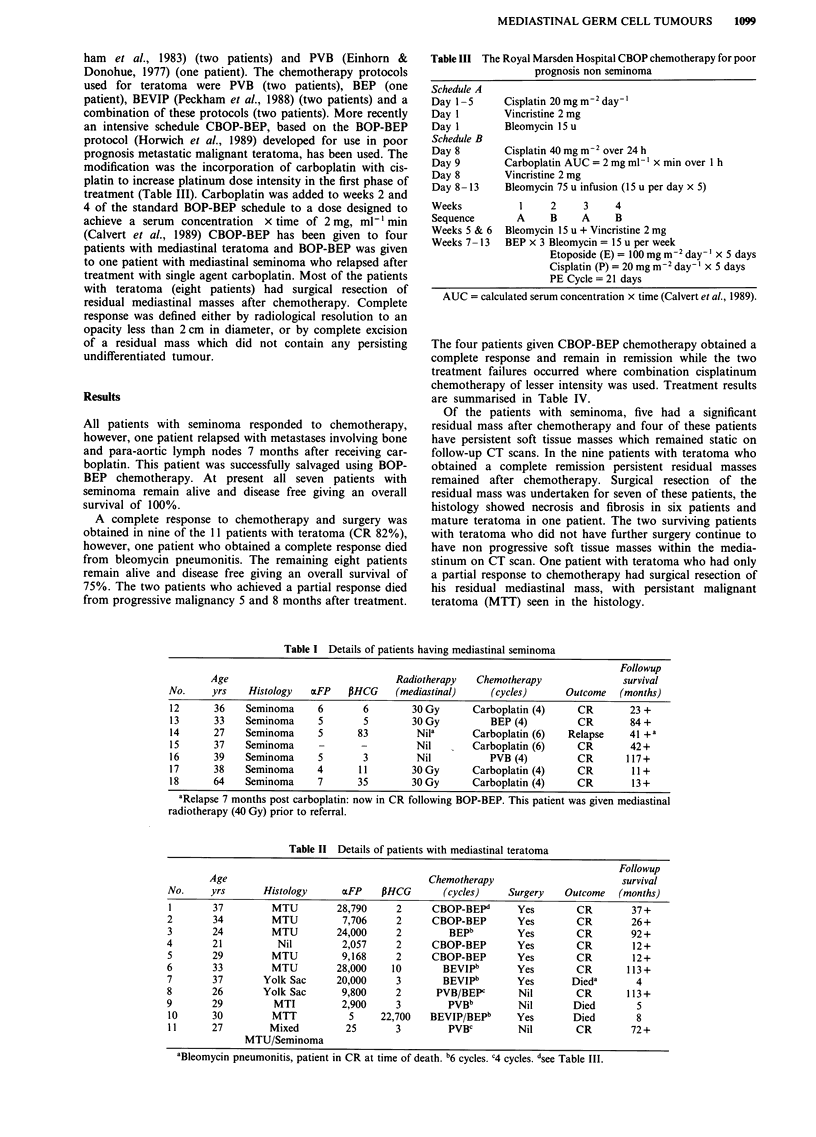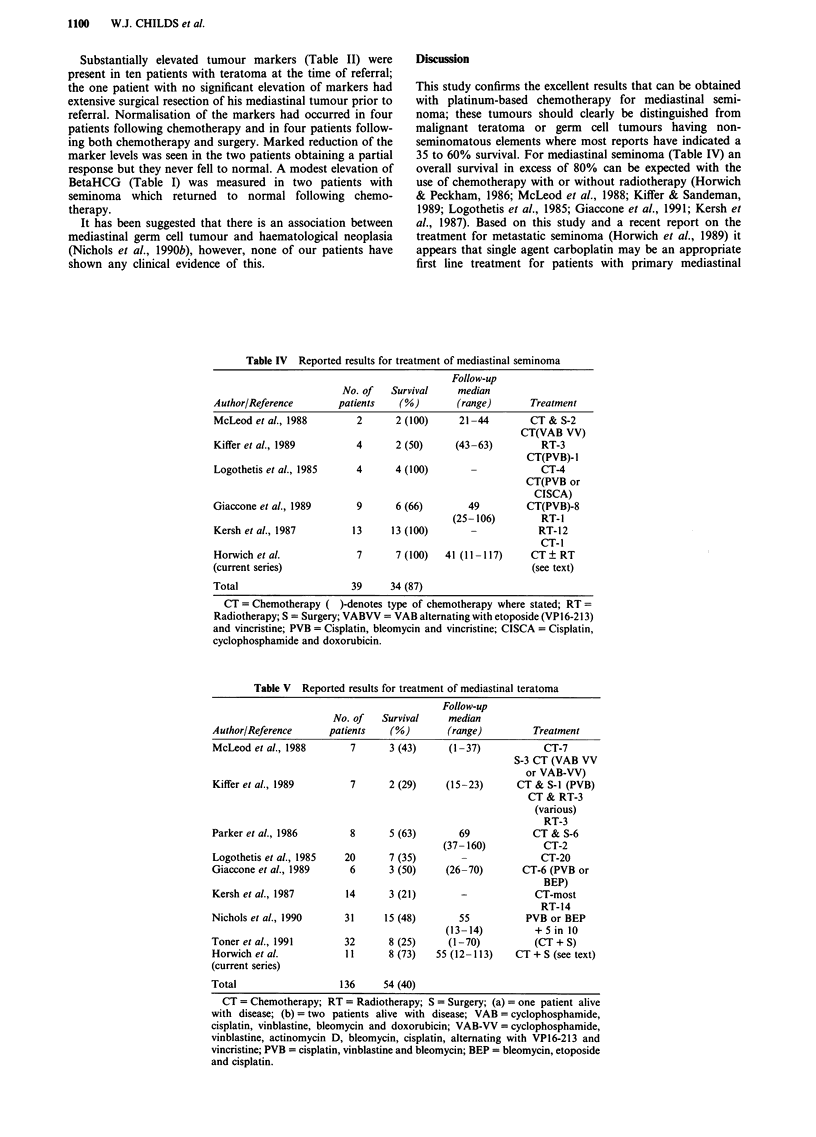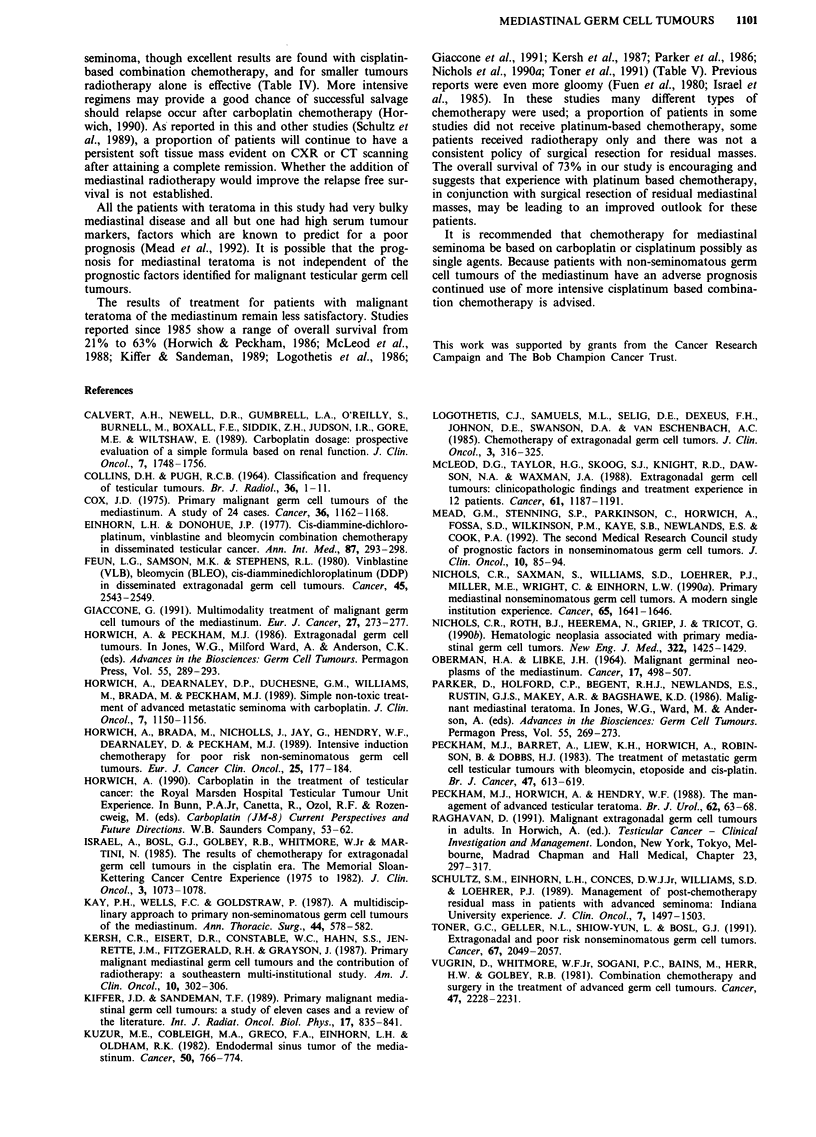# Primary malignant mediastinal germ cell tumours: improved prognosis with platinum-based chemotherapy and surgery.

**DOI:** 10.1038/bjc.1993.201

**Published:** 1993-05

**Authors:** W. J. Childs, P. Goldstraw, J. E. Nicholls, D. P. Dearnaley, A. Horwich

**Affiliations:** Oncology Centre, Auckland Hospital, New Zealand.

## Abstract

A retrospective analysis was performed of 18 patients with primary malignant germ cell tumours of the mediastinum treated with platinum-based chemotherapy between 1977 and 1990. All seven patients with pure seminoma were treated initially with chemotherapy and four of these patients received additional mediastinal radiotherapy. Only one patient relapsed; his initial therapy had included radiotherapy and single-agent carboplatin and he was successfully salvaged with combination chemotherapy. With a follow-up of 11 to 117 months (median 41 months) all seven patients with seminoma remain alive and disease free giving an overall survival of 100%. Eleven patients had malignant non seminoma; following chemotherapy eight of these had elective surgical resection of residual mediastinal masses. Complete remission was achieved in nine (82%) patients, however, one of these patients died from bleomycin pneumonitis. With a follow-up of 12 to 113 months (median 55 months) eight of 11 (73%) patients with malignant mediastinal teratoma remain alive and disease free.


*Br. J. Cancer* (1993), **67**, 1098–1101 © Macmillan Press Ltd., 1993

### Primary malignant mediastinal germ cell tumours: improved prognosis with platinum-based chemotherapy and surgery

W.J. Childs[1], P. Goldstraw[2], J.E. Nicholls[3], D.P. Dearnaley[3] & A. Horwich[3]

[1]*Oncology Centre, Auckland Hospital, Park Road, Auckland, New Zealand;* [2]*The Royal Brompton Hospital, Fulham Road, London SW3 6JJ;* [3]*Academic Unit, The Royal Marsden Hospital, Downs Road, Sutton, Surrey SM2 5PT, UK.*

**Summary** A retrospective analysis was performed of 18 patients with primary malignant germ cell tumours of the mediastinum treated with platinum-based chemotherapy between 1977 and 1990. All seven patients with pure seminoma were treated initially with chemotherapy and four of these patients received additional mediastinal radiotherapy. Only one patient relapsed; his initial therapy had included radiotherapy and single-agent carboplatin and he was successfully salvaged with combination chemotherapy. With a follow-up of 11 to 117 months (median 41 months) all seven patients with seminoma remain alive and disease free giving an overall survival of 100%. Eleven patients had malignant non seminoma; following chemotherapy eight of these had elective surgical resection of residual mediastinal masses. Complete remission was achieved in nine (82%) patients, however, one of these patients died from bleomycin pneumonitis. With a follow-up of 12 to 113 months (median 55 months) eight of 11 (73%) patients with malignant mediastinal teratoma remain alive and disease free.

Although rare, primary malignant germ cell tumours of the mediastinum are a clinically important group of tumours which account for 5 to 13% of all malignant mediastinal tumours (Oberman & Libke, 1964; Cox, 1975; Raghaven, 1991). It is estimated that 1 to 4% of all primary malignant germ cell tumours arise from an extragonadal site (Collins & Pugh, 1964) of which the anterior mediastinum is the second most common. Despite having a spectrum of histology similar to primary gonadal tumours it has been reported that they have a worse prognosis compared to metastatic germ cell tumours of gonadal origin (Kuzur *et al.*, 1982; Feun *et al.*, 1980; Toner *et al.*, 1991; Vugrin *et al.*, 1981). This certainly appears to be true for mediastinal teratoma, however, most reports now show that good results can be obtained from using platinum based chemotherapy for primary mediastinal seminoma (Israel *et al.*, 1985). The current prognosis of mediastinal teratoma has been difficult to assess because many reported series extend back over a long time period. We have restricted this analysis to the era of platinum based chemotherapy and have assessed all patients referred to the Testicular Tumour Unit at the Royal Marsden Hospital with primary malignant germ cell tumours of the mediastinum between 1977 and 1989. Results on several of these patients have been reported elsewhere (Horwich *et al.*, 1986; Kay *et al.*, 1987).

### Patients details and methods

The study was a retrospective review of all case notes of patients with the diagnosis of primary malignant mediastinal germ cell tumour who had received platinum based chemotherapy at the RMH between 1977 and 1989. The criteria for inclusion were that patients had to present with a mass in the anterior mediastinum, have histology or marker evidence of a germ cell tumour; and have normal testes on initial and subsequent examinations. The distinction from occult testicular primary cancer was based on the location of the mass within the anterior mediastinum, since this is an extremely uncommon metastatic site from overt testicular primaries. Patients who had received previous chemotherapy were excluded. Four patients with seminoma and six with non-seminoma were reported previously (Horwich *et al.*, 1986), but the follow-up data has been extended.

Correspondence: A. Horwich, Department of Radiotherapy and Oncology, The Institute of Cancer Research and The Royal Marsden Hospital, Downs Road, Sutton, Surrey SM2 5PT, UK.
Received 18 September 1992; and in revised form 20 December 1992.

### *Patient characteristics*

The patients were all male and had a median age of 33 years (range: 21 to 64 years). A biopsy of the tumour was obtained prior to treatment in all but one patient, seven patients had pure seminoma and 11 had malignant teratoma. The one patient who did not have a biopsy was an 18 year old with a larger anterior mediastinal mass and an increased serum alphafetoprotein of 2,057 $\mathrm{Iu\,l^{-1}}$. The most common presenting clinical features were dyspnoea (50%), cough (50%), chest pain (67%) and constitutional symptoms (lethargy, malaise, anorexia or weight loss) (39%). The tumours were arising in the anterior mediastinum and were very extensive in all patients. Direct invasion of tumour into the anterior chest wall was present in four patients with seminoma. Metastases outside the mediastinum were found in three patients (27%) with teratoma and four patients (57%) with seminoma. The sites of metastases were axilla or neck nodes (five patients), lung (two patients), bone (one patient) and upper para-aortic nodes (one patient).

### *Investigations*

On referral to the Royal Marsden Hospital all patients had a chest X-Ray, full blood count, liver function and sequential measurement of serum alphafetoprotein (AFP) and Beta subunit of human chorionic gonadotrophin (HCG). Most had a CT scan of the chest (15 patients) and abdomen (14 patients). An ultrasound scan of the testes was carried out in eight patients. Other investigations such as lymphangiography (four patients), whole lung tomography (three patients), bone scintigraphy (two patients) or abdominal ultrasound were performed where indicated or when CT scanning was unavailable.

### *Previous treatment*

Three patients had received some treatment prior to referral (surgery two, radiotherapy one). Two of these patients had persistent tumour after this initial treatment and one patient had relapsed after an apparent complete response.

### *Treatment*

Details of treatment given to each patient are outlined in Tables I and II. The usual management for patients with seminoma was single agent carboplatin (Horwich *et al.*, 1989) (five patients) followed by mediastinal radiotherapy to a total midplane dose of 30 Gy in 15 fractions over 3 weeks. Other chemotherapy protocols used for seminoma were BEP (Peckham *et al.*, 1983) (two patients) and PVB (Einhorn & Donohue, 1977) (one patient). The chemotherapy protocols used for teratoma were PVB (two patients), BEP (one patient), BEVIP (Peckham *et al.*, 1988) (two patients) and a combination of these protocols (two patients). More recently an intensive schedule CBOP-BEP, based on the BOP-BEP protocol (Horwich *et al.*, 1989) developed for use in poor prognosis metastatic malignant teratoma, has been used. The modification was the incorporation of carboplatin with cisplatin to increase platinum dose intensity in the first phase of treatment (Table III). Carboplatin was added to weeks 2 and 4 of the standard BOP-BEP schedule to a dose designed to achieve a serum concentration × time of 2 mg, $ml^{-1}$ min (Calvert *et al.*, 1989) CBOP-BEP has been given to four patients with mediastinal teratoma and BOP-BEP was given to one patient with mediastinal seminoma who relapsed after treatment with single agent carboplatin. Most of the patients with teratoma (eight patients) had surgical resection of residual mediastinal masses after chemotherapy. Complete response was defined either by radiological resolution to an opacity less than 2 cm in diameter, or by complete excision of a residual mass which did not contain any persisting undifferentiated tumour.

### Results

All patients with seminoma responded to chemotherapy, however, one patient relapsed with metastases involving bone and para-aortic lymph nodes 7 months after receiving carboplatin. This patient was successfully salvaged using BOP-BEP chemotherapy. At present all seven patients with seminoma remain alive and disease free giving an overall survival of 100%.

A complete response to chemotherapy and surgery was obtained in nine of the 11 patients with teratoma (CR 82%), however, one patient who obtained a complete response died from bleomycin pneumonitis. The remaining eight patients remain alive and disease free giving an overall survival of 75%. The two patients who achieved a partial response died from progressive malignancy 5 and 8 months after treatment.

**Table III** The Royal Marsden Hospital CBOP chemotherapy for poor prognosis non seminoma

| | |
|---|---|
| *Schedule A* | |
| Day 1–5 | Cisplatin 20 mg $m^{-2}$ $day^{-1}$ |
| Day 1 | Vincristine 2 mg |
| Day 1 | Bleomycin 15 u |
| *Schedule B* | |
| Day 8 | Cisplatin 40 mg $m^{-2}$ over 24 h |
| Day 9 | Carboplatin AUC = 2 mg $ml^{-1}$ × min over 1 h |
| Day 8 | Vincristine 2 mg |
| Day 8–13 | Bleomycin 75 u infusion (15 u per day × 5) |
| Weeks | 1 2 3 4 |
| Sequence | A B A B |
| Weeks 5 & 6 | Bleomycin 15 u + Vincristine 2 mg |
| Weeks 7–13 | BEP × 3 Bleomycin = 15 u per week |
| | Etoposide (E) = 100 mg $m^{-2}$ $day^{-1}$ × 5 days |
| | Cisplatin (P) = 20 mg $m^{-2}$ $day^{-1}$ × 5 days |
| | PE Cycle = 21 days |

AUC = calculated serum concentration × time (Calvert *et al.*, 1989).

The four patients given CBOP-BEP chemotherapy obtained a complete response and remain in remission while the two treatment failures occurred where combination cisplatinum chemotherapy of lesser intensity was used. Treatment results are summarised in Table IV.

Of the patients with seminoma, five had a significant residual mass after chemotherapy and four of these patients have persistent soft tissue masses which remained static on follow-up CT scans. In the nine patients with teratoma who obtained a complete remission persistent residual masses remained after chemotherapy. Surgical resection of the residual mass was undertaken for seven of these patients, the histology showed necrosis and fibrosis in six patients and mature teratoma in one patient. The two surviving patients with teratoma who did not have further surgery continue to have non progressive soft tissue masses within the mediastinum on CT scan. One patient with teratoma who had only a partial response to chemotherapy had surgical resection of his residual mediastinal mass, with persistant malignant teratoma (MTT) seen in the histology.

**Table I** Details of patients having mediastinal seminoma

| *No.* | *Age yrs* | *Histology* | *αFP* | *βHCG* | *Radiotherapy (mediastinal)* | *Chemotherapy (cycles)* | *Outcome* | *Followup survival (months)* |
|---|---|---|---|---|---|---|---|---|
| 12 | 36 | Seminoma | 6 | 6 | 30 Gy | Carboplatin (4) | CR | 23 + |
| 13 | 33 | Seminoma | 5 | 5 | 30 Gy | BEP (4) | CR | 84 + |
| 14 | 27 | Seminoma | 5 | 83 | Nil[a] | Carboplatin (6) | Relapse | 41 +[a] |
| 15 | 37 | Seminoma | – | – | Nil | Carboplatin (6) | CR | 42+ |
| 16 | 39 | Seminoma | 5 | 3 | Nil | PVB (4) | CR | 117+ |
| 17 | 38 | Seminoma | 4 | 11 | 30 Gy | Carboplatin (4) | CR | 11+ |
| 18 | 64 | Seminoma | 7 | 35 | 30 Gy | Carboplatin (4) | CR | 13+ |

[a]Relapse 7 months post carboplatin: now in CR following BOP-BEP. This patient was given mediastinal radiotherapy (40 Gy) prior to referral.

**Table II** Details of patients with mediastinal teratoma

| *No.* | *Age yrs* | *Histology* | *αFP* | *βHCG* | *Chemotherapy (cycles)* | *Surgery* | *Outcome* | *Followup survival (months)* |
|---|---|---|---|---|---|---|---|---|
| 1 | 37 | MTU | 28,790 | 2 | CBOP-BEP[d] | Yes | CR | 37+ |
| 2 | 34 | MTU | 7,706 | 2 | CBOP-BEP | Yes | CR | 26+ |
| 3 | 24 | MTU | 24,000 | 2 | BEP[b] | Yes | CR | 92+ |
| 4 | 21 | Nil | 2,057 | 2 | CBOP-BEP | Yes | CR | 12+ |
| 5 | 29 | MTU | 9,168 | 2 | CBOP-BEP | Yes | CR | 12+ |
| 6 | 33 | MTU | 28,000 | 10 | BEVIP[b] | Yes | CR | 113+ |
| 7 | 37 | Yolk Sac | 20,000 | 3 | BEVIP[b] | Yes | Died[a] | 4 |
| 8 | 26 | Yolk Sac | 9,800 | 2 | PVB/BEP[c] | Nil | CR | 113+ |
| 9 | 29 | MTI | 2,900 | 3 | PVB[b] | Nil | Died | 5 |
| 10 | 30 | MTT | 5 | 22,700 | BEVIP/BEP[b] | Yes | Died | 8 |
| 11 | 27 | Mixed MTU/Seminoma | 25 | 3 | PVB[c] | Nil | CR | 72+ |

[a]Bleomycin pneumonitis, patient in CR at time of death. [b]6 cycles. [c]4 cycles. [d]see Table III.

Substantially elevated tumour markers (Table II) were present in ten patients with teratoma at the time of referral; the one patient with no significant elevation of markers had extensive surgical resection of his mediastinal tumour prior to referral. Normalisation of the markers had occurred in four patients following chemotherapy and in four patients following both chemotherapy and surgery. Marked reduction of the marker levels was seen in the two patients obtaining a partial response but they never fell to normal. A modest elevation of BetaHCG (Table I) was measured in two patients with seminoma which returned to normal following chemotherapy.

It has been suggested that there is an association between mediastinal germ cell tumour and haematological neoplasia (Nichols *et al.*, 1990*b*), however, none of our patients have shown any clinical evidence of this.

### Discussion

This study confirms the excellent results that can be obtained with platinum-based chemotherapy for mediastinal seminoma; these tumours should clearly be distinguished from malignant teratoma or germ cell tumours having non-seminomatous elements where most reports have indicated a 35 to 60% survival. For mediastinal seminoma (Table IV) an overall survival in excess of 80% can be expected with the use of chemotherapy with or without radiotherapy (Horwich & Peckham, 1986; McLeod *et al.*, 1988; Kiffer & Sandeman, 1989; Logothetis *et al.*, 1985; Giaccone *et al.*, 1991; Kersh *et al.*, 1987). Based on this study and a recent report on the treatment for metastatic seminoma (Horwich *et al.*, 1989) it appears that single agent carboplatin may be an appropriate first line treatment for patients with primary mediastinal

**Table IV** Reported results for treatment of mediastinal seminoma

| *Author/Reference* | *No. of patients* | *Survival (%)* | *Follow-up median (range)* | *Treatment* |
|---|---|---|---|---|
| McLeod *et al.*, 1988 | 2 | 2 (100) | 21-44 | CT & S-2<br>CT(VAB VV) |
| Kiffer *et al.*, 1989 | 4 | 2 (50) | (43-63) | RT-3<br>CT(PVB)-1 |
| Logothetis *et al.*, 1985 | 4 | 4 (100) | - | CT-4<br>CT(PVB or CISCA) |
| Giaccone *et al.*, 1989 | 9 | 6 (66) | 49<br>(25-106) | CT(PVB)-8<br>RT-1 |
| Kersh *et al.*, 1987 | 13 | 13 (100) | - | RT-12<br>CT-1 |
| Horwich *et al.* (current series) | 7 | 7 (100) | 41 (11-117) | CT ± RT (see text) |
| Total | 39 | 34 (87) | | |

CT = Chemotherapy ( )-denotes type of chemotherapy where stated; RT = Radiotherapy; S = Surgery; VABVV = VAB alternating with etoposide (VP16-213) and vincristine; PVB = Cisplatin, bleomycin and vincristine; CISCA = Cisplatin, cyclophosphamide and doxorubicin.

**Table V** Reported results for treatment of mediastinal teratoma

| *Author/Reference* | *No. of patients* | *Survival (%)* | *Follow-up median (range)* | *Treatment* |
|---|---|---|---|---|
| McLeod *et al.*, 1988 | 7 | 3 (43) | (1-37) | CT-7<br>S-3 CT (VAB VV or VAB-VV) |
| Kiffer *et al.*, 1989 | 7 | 2 (29) | (15-23) | CT & S-1 (PVB)<br>CT & RT-3 (various)<br>RT-3 |
| Parker *et al.*, 1986 | 8 | 5 (63) | 69<br>(37-160) | CT & S-6<br>CT-2 |
| Logothetis *et al.*, 1985 | 20 | 7 (35) | - | CT-20 |
| Giaccone *et al.*, 1989 | 6 | 3 (50) | (26-70) | CT-6 (PVB or BEP) |
| Kersh *et al.*, 1987 | 14 | 3 (21) | - | CT-most<br>RT-14 |
| Nichols *et al.*, 1990 | 31 | 15 (48) | 55<br>(13-14) | PVB or BEP<br>+ 5 in 10 |
| Toner *et al.*, 1991 | 32 | 8 (25) | (1-70) | (CT + S) |
| Horwich *et al.* (current series) | 11 | 8 (73) | 55 (12-113) | CT + S (see text) |
| Total | 136 | 54 (40) | | |

CT = Chemotherapy; RT = Radiotherapy; S = Surgery; (a) = one patient alive with disease; (b) = two patients alive with disease; VAB = cyclophosphamide, cisplatin, vinblastine, bleomycin and doxorubicin; VAB-VV = cyclophosphamide, vinblastine, actinomycin D, bleomycin, cisplatin, alternating with VP16-213 and vincristine; PVB = cisplatin, vinblastine and bleomycin; BEP = bleomycin, etoposide and cisplatin.

seminoma, though excellent results are found with cisplatin-based combination chemotherapy, and for smaller tumours radiotherapy alone is effective (Table IV). More intensive regimens may provide a good chance of successful salvage should relapse occur after carboplatin chemotherapy (Horwich, 1990). As reported in this and other studies (Schultz *et al.*, 1989), a proportion of patients will continue to have a persistent soft tissue mass evident on CXR or CT scanning after attaining a complete remission. Whether the addition of mediastinal radiotherapy would improve the relapse free survival is not established.

All the patients with teratoma in this study had very bulky mediastinal disease and all but one had high serum tumour markers, factors which are known to predict for a poor prognosis (Mead *et al.*, 1992). It is possible that the prognosis for mediastinal teratoma is not independent of the prognostic factors identified for malignant testicular germ cell tumours.

The results of treatment for patients with malignant teratoma of the mediastinum remain less satisfactory. Studies reported since 1985 show a range of overall survival from 21% to 63% (Horwich & Peckham, 1986; McLeod *et al.*, 1988; Kiffer & Sandeman, 1989; Logothetis *et al.*, 1986; Giaccone *et al.*, 1991; Kersh *et al.*, 1987; Parker *et al.*, 1986; Nichols *et al.*, 1990*a*; Toner *et al.*, 1991) (Table V). Previous reports were even more gloomy (Fuen *et al.*, 1980; Israel *et al.*, 1985). In these studies many different types of chemotherapy were used; a proportion of patients in some studies did not receive platinum-based chemotherapy, some patients received radiotherapy only and there was not a consistent policy of surgical resection for residual masses. The overall survival of 73% in our study is encouraging and suggests that experience with platinum based chemotherapy, in conjunction with surgical resection of residual mediastinal masses, may be leading to an improved outlook for these patients.

It is recommended that chemotherapy for mediastinal seminoma be based on carboplatin or cisplatinum possibly as single agents. Because patients with non-seminomatous germ cell tumours of the mediastinum have an adverse prognosis continued use of more intensive cisplatinum based combination chemotherapy is advised.

This work was supported by grants from the Cancer Research Campaign and The Bob Champion Cancer Trust.

### References

CALVERT, A.H., NEWELL, D.R., GUMBRELL, L.A., O'REILLY, S., BURNELL, M., BOXALL, F.E., SIDDIK, Z.H., JUDSON, I.R., GORE, M.E. & WILTSHAW, E. (1989). Carboplatin dosage: prospective evaluation of a simple formula based on renal function. *J. Clin. Oncol.*, **7**, 1748–1756.

COLLINS, D.H. & PUGH, R.C.B. (1964). Classification and frequency of testicular tumours. *Br. J. Radiol.*, **36**, 1–11.

COX, J.D. (1975). Primary malignant germ cell tumours of the mediastinum. A study of 24 cases. *Cancer*, **36**, 1162–1168.

EINHORN, L.H. & DONOHUE, J.P. (1977). Cis-diammine-dichloroplatinum, vinblastine and bleomycin combination chemotherapy in disseminated testicular cancer. *Ann. Int. Med.*, **87**, 293–298.

FEUN, L.G., SAMSON, M.K. & STEPHENS, R.L. (1980). Vinblastine (VLB), bleomycin (BLEO), cis-diamminedichloroplatinum (DDP) in disseminated extragonadal germ cell tumours. *Cancer*, **45**, 2543–2549.

GIACCONE, G. (1991). Multimodality treatment of malignant germ cell tumours of the mediastinum. *Eur. J. Cancer*, **27**, 273–277.

HORWICH, A. & PECKHAM, M.J. (1986). Extragonadal germ cell tumours. In Jones, W.G., Milford Ward, A. & Anderson, C.K. (eds). *Advances in the Biosciences: Germ Cell Tumours*. Permagon Press, Vol. 55, 289–293.

HORWICH, A., DEARNALEY, D.P., DUCHESNE, G.M., WILLIAMS, M., BRADA, M. & PECKHAM, M.J. (1989). Simple non-toxic treatment of advanced metastatic seminoma with carboplatin. *J. Clin. Oncol.*, **7**, 1150–1156.

HORWICH, A., BRADA, M., NICHOLLS, J., JAY, G., HENDRY, W.F., DEARNALEY, D. & PECKHAM, M.J. (1989). Intensive induction chemotherapy for poor risk non-seminomatous germ cell tumours. *Eur. J. Cancer Clin. Oncol.*, **25**, 177–184.

HORWICH, A. (1990). Carboplatin in the treatment of testicular cancer: the Royal Marsden Hospital Testicular Tumour Unit Experience. In Bunn, P.A.Jr, Canetta, R., Ozol, R.F. & Rozencweig, M. (eds). *Carboplatin (JM-8) Current Perspectives and Future Directions*. W.B. Saunders Company, 53–62.

ISRAEL, A., BOSL, G.J., GOLBEY, R.B., WHITMORE, W.Jr & MARTINI, N. (1985). The results of chemotherapy for extragonadal germ cell tumours in the cisplatin era. The Memorial Sloan-Kettering Cancer Centre Experience (1975 to 1982). *J. Clin. Oncol.*, **3**, 1073–1078.

KAY, P.H., WELLS, F.C. & GOLDSTRAW, P. (1987). A multidisciplinary approach to primary non-seminomatous germ cell tumours of the mediastinum. *Ann. Thoracic. Surg.*, **44**, 578–582.

KERSH, C.R., EISERT, D.R., CONSTABLE, W.C., HAHN, S.S., JENRETTE, J.M., FITZGERALD, R.H. & GRAYSON, J. (1987). Primary malignant mediastinal germ cell tumours and the contribution of radiotherapy: a southeastern multi-institutional study. *Am. J. Clin. Oncol.*, **10**, 302–306.

KIFFER, J.D. & SANDEMAN, T.F. (1989). Primary malignant mediastinal germ cell tumours: a study of eleven cases and a review of the literature. *Int. J. Radiat. Oncol. Biol. Phys.*, **17**, 835–841.

KUZUR, M.E., COBLEIGH, M.A., GRECO, F.A., EINHORN, L.H. & OLDHAM, R.K. (1982). Endodermal sinus tumor of the mediastinum. *Cancer*, **50**, 766–774.

LOGOTHETIS, C.J., SAMUELS, M.L., SELIG, D.E., DEXEUS, F.H., JOHNON, D.E., SWANSON, D.A. & VAN ESCHENBACH, A.C. (1985). Chemotherapy of extragonadal germ cell tumors. *J. Clin. Oncol.*, **3**, 316–325.

McLEOD, D.G., TAYLOR, H.G., SKOOG, S.J., KNIGHT, R.D., DAWSON, N.A. & WAXMAN, J.A. (1988). Extragonadal germ cell tumours: clinicopathologic findings and treatment experience in 12 patients. *Cancer*, **61**, 1187–1191.

MEAD, G.M., STENNING, S.P., PARKINSON, C., HORWICH, A., FOSSA, S.D., WILKINSON, P.M., KAYE, S.B., NEWLANDS, E.S. & COOK, P.A. (1992). The second Medical Research Council study of prognostic factors in nonseminomatous germ cell tumors. *J. Clin. Oncol.*, **10**, 85–94.

NICHOLS, C.R., SAXMAN, S., WILLIAMS, S.D., LOEHRER, P.J., MILLER, M.E., WRIGHT, C. & EINHORN, L.W. (1990*a*). Primary mediastinal nonseminomatous germ cell tumors. A modern single institution experience. *Cancer*, **65**, 1641–1646.

NICHOLS, C.R., ROTH, B.J., HEEREMA, N., GRIEP, J. & TRICOT, G. (1990*b*). Hematologic neoplasia associated with primary mediastinal germ cell tumors. *New Eng. J. Med.*, **322**, 1425–1429.

OBERMAN, H.A. & LIBKE, J.H. (1964). Malignant germinal neoplasms of the mediastinum. *Cancer*, **17**, 498–507.

PARKER, D., HOLFORD, C.P., BEGENT, R.H.J., NEWLANDS, E.S., RUSTIN, G.J.S., MAKEY, A.R. & BAGSHAWE, K.D. (1986). Malignant mediastinal teratoma. In Jones, W.G., Ward, M. & Anderson, A. (eds). *Advances in the Biosciences: Germ Cell Tumours*. Permagon Press, Vol. 55, 269–273.

PECKHAM, M.J., BARRET, A., LIEW, K.H., HORWICH, A., ROBINSON, B. & DOBBS, H.J. (1983). The treatment of metastatic germ cell testicular tumours with bleomycin, etoposide and cis-platin. *Br. J. Cancer*, **47**, 613–619.

PECKHAM, M.J., HORWICH, A. & HENDRY, W.F. (1988). The management of advanced testicular teratoma. *Br. J. Urol.*, **62**, 63–68.

RAGHAVAN, D. (1991). Malignant extragonadal germ cell tumours in adults. In Horwich, A. (ed.). *Testicular Cancer – Clinical Investigation and Management*. London, New York, Tokyo, Melbourne, Madrad Chapman and Hall Medical, Chapter 23, 297–317.

SCHULTZ, S.M., EINHORN, L.H., CONCES, D.W.J.Jr, WILLIAMS, S.D. & LOEHRER, P.J. (1989). Management of post-chemotherapy residual mass in patients with advanced seminoma: Indiana University experience. *J. Clin. Oncol.*, **7**, 1497–1503.

TONER, G.C., GELLER, N.L., SHIOW-YUN, L. & BOSL, G.J. (1991). Extragonadal and poor risk nonseminomatous germ cell tumors. *Cancer*, **67**, 2049–2057.

VUGRIN, D., WHITMORE, W.F.Jr, SOGANI, P.C., BAINS, M., HERR, H.W. & GOLBEY, R.B. (1981). Combination chemotherapy and surgery in the treatment of advanced germ cell tumours. *Cancer*, **47**, 2228–2231.